# Digital hazards for feeding and eating: what we know and what we don’t

**DOI:** 10.1007/s11920-021-01271-7

**Published:** 2021-07-15

**Authors:** Konstantinos Ioannidis, Samuel R Chamberlain

**Affiliations:** a Department of Psychiatry, University of Cambridge, UK; b Cambridge and Peterborough NHS Foundation Trust, Cambridge, UK; c Department of International Health, Care and Public Health Research Institute, Maastricht University, Maastricht, Netherlands; d Department of Psychiatry, University of Southampton, UK; e Southern Health NHS Foundation Trust, Southampton, UK

**Keywords:** anorexia nervosa, bulimia nervosa, binge eating disorder, eating disorder, internet addiction, social networking site, social media, problematic internet use

## Abstract

**Purpose of review:**

We aimed to accrue recent evidence exploring effects of modern online activities (e.g. Internet use) on feeding and eating disorder symptoms, and related traits. We examined available evidence to ascertain any direct influences from online activities on feeding and eating disorders, thereby shedding light on putative mechanisms by which those influences may occur.

**Recent findings:**

Many facets of problematic usage of the internet correlate cross sectionally with eating disorder and related psychopathology. There is evidence to suggest that significant effects do exist in the direction of specific internet activities contributing to eating disorder symptoms, viewed dimensionally. Putative mechanisms are discussed. However a significant number of eating disorder phenotypes and internet-related activities remain under-researched.

**Summary:**

Specific facets of engagement with the online environment appear to confer risk for feeding and eating problems, evidence being strongest for non-clinical studies using dimensional measures. More research is required to rigorously confirm causal effects, including in patients meeting formal diagnostic criteria for eating disorders. We also highlight the need for high quality evidence to explore how eating disorder phenotypes are commonly as well as uniquely affected by different online activities. Such research is needed in order that scientific understanding in this area can be translated to protect those most at risk of disordered eating, including through changes in public health approaches and clinical practice.

## Introduction

Eating disorders (EDs) confer an important health burden for societies worldwide (1,2). Anorexia nervosa (AN) has the highest morbidity and mortality of all mental illnesses (3) and a significant lifetime prevalence, depending on diagnostic criteria and population under study (4). Bulimia nervosa (BN), binge eating disorder (BED) (5) and other less studied known diagnostic categories e.g. avoidant-restrictive food intake disorder (ARFID) are considered more common and are often underdiagnosed (6). Restrictive eating, typical of AN, is often interspersed with binge-eating or purging or excessive exercise behaviors and cross-diagnostic interplay of symptoms is very common (7), while more often than not, individuals swap between ED diagnostic classifications during their lifetime. Specifically, since the introduction of Diagnostic and Statistical Manual Version 5 (DSM-5) criteria for feeding and eating disorders (8), the prevalence of diagnostic groups is considered to have increased among all main diagnostic categories (9). Eating disorders have a complex partially known pathophysiology which implicates multiple layers of socio-cultural and biological contextual variables (7). There is paucity of experimental research in the field (10) and most things we know about the pathophysiology of EDs, derive from ‘quasi-experimental’ studies and randomized-controlled trials (RCTs). While RCTs include a manipulated component that separates the intervention and the control arm, often several factors are targeted simultaneously through comprehensive treatment programs; this approach does not necessarily allow for the identification of causal effects with adequate enough precision to specify effects to fine detail (10). While we have been building our understanding of the gene × environment interaction to identify risk factors for EDs towards the end of the 20^th^ century, efforts were made through observational research to understand the influences of popular media (paper media, advertisements, TV) on eating disorder risk factors. Pooled evidence suggested that women in particular are suffering from a hazardous influence from exposure to popular media in terms of body dissatisfaction, internalization of thinness ideals and disordered eating (11,12). Exposure to beauty ideals coupled with an internalization of media mandates led to a socio-cultural body-ideal mismatch. The latter has been considered a piece of the puzzle in the gene × environment interaction driving ED pathophysiology (13).

In the 1980s, a mighty force was born: the world-wide-web. It went from a whimsical idea to dominating real life communication, entertainment and work in less than four decades, and has now shaped our lives irreversibly. While the essential and extremely valuable applications of the internet in daily life are irrefutable, maladaptive forms of engagement with the online environment, encompassing a variety of activities (e.g. overuse of social media, streaming media, gaming, gambling, pornography (14•)) has been associated with marked functional impairment (15,16). The term Problematic usage of the internet (PUI) was coined to describe such dysfunctional engagement with multiple facets of internet based activities (16••). PUI is now linked with poorer health, worse social, vocational or academic outcomes or lower quality of life (16–18). Since 2010, and given the previously mentioned concerning influences of media on eating disorder outcomes, significant concern arose within researchers, clinicians and carers about those suffering from eating disorders, as to whether specific engagement with online content would impact on the development, course and management of eating disorders across the life span. A number of observational and experimental studies were performed since in attempt to disentangle effects and described the relevant relationships (19••).

## New and interesting findings

Research in the last decade started by exploring the membership in pro-anorexia forums (consumption and engagement in “Pro-ANA” web based content) (20) as those became very popular amongst anorexia sufferers. A few important steps towards understanding the role of PUI in ED pathophysiology were made, when Facebook use was found to prospectively predict an increase in drive for thinness two years later (21••), whereas receiving negative feedback through Facebook interactions was found to negatively associate with disordered eating attitudes (22). Social networking site (SNS) use also predicted an increase in body dissatisfaction (23). These results supported a causal hypothesis of the role that social media may have in eating disorders. However, such effects of SNS on ED were not identified in other studies (24). Being exposed to pro-ED content or “fitspiration” content (media content aimed to inspire towards fitness activity or a fit body ideal) or regular popular social media (which often contain dieting, fitness or other appearance focused content) as opposed to neutral conditions (e.g. viewing travel images or spending time reading encyclopedia entries online) seems to bear a degree of risk towards the development of mood and self-esteem difficulties that fuels EDs. Those experimental conditions were tested to show the ‘PUI condition’ associating stronger with lower appearance self-esteem (25), higher ED symptomatology (26–29); those with appearance comparison traits were more prone to low mood effects from exposure to social media (30,31). Another important aspect of engaging with the online content is the exposure to manipulated content, as this has the potential of creating unrealistic expectations of appearance and beauty. Under experimental conditions, self-photo editing led to increased negative mood and body (facial) dissatisfaction, with higher levels of editing leading to more dissatisfaction. Furthermore, enhancement-free images seem to have less effect on body dissatisfaction, suggesting that self-photo manipulation may be playing a role in body dissatisfaction effects (32•,^33^). While PUI x EDs interactions have been more explored in adult participants under experimental conditions, particularly AN and BN are considered to sometimes have their beginnings in adolescence or earlier in life. The effects of appearance focused gaming vs. ED-neutral gaming were assessed in young girls: those exposed to appearance focused gaming had higher body dissatisfaction (34•). This essentially represents a form of replication work from physical world experiments to the online environment; pre-pubertal girls exposed to Barbie doll play have high internalized thin ideals (35) and reduced food intake (36); appearance focused online gaming may influence EDs through a mechanistically similar fashion. This is a critically important point while online gaming in children and adolescents is growing as a cultural norm in developed societies, in which EDs are also more prevalent.

While activity tracking apps and fitness apps have become very popular amongst the eating disorder community, problematic usage of the internet was shown to partially mediate the relationship between sensation seeking impulsivity and eating disorders with high exercise levels (37). Additionally, problematic usage of the internet was found to partially mediate the link between obsessionality and eating disorders with high exercise levels (37). Usage of fitness and calorie tracking apps have correlated with eating disorder and related psychopathology both in males and females (38–40). It is possible that in the background of particular latent traits of impulsivity or obsessionality/compulsivity, exposure to calorie or fitness apps may trigger problematic behaviors (e.g. excessive exercise) and potentially contribute to the development of clinically significant levels of ED in those who are vulnerable. However, those are outcomes of online surveys with various methodological limitations and disallow the drawing of any causal inferences.

Another developing area of interest has been the investigation of how dating sites may be influencing eating disorder and related psychopathology. Dating sites put emphasis on physical appearance and sharing of image based content. As such, the individual is invited to judge others by their image while eliciting judgment on themselves. By creating this content the individual engages in a process known as self-objectification (41), however, self-objectification may also occur in other forms of social and popular media. Self-objectification is moderately correlated with disordered eating in meta-analysis (Pearson’s r=0.39, 53 cross-sectional studies), with larger effect sizes identified in females (42••). Dating sites use has been cross-sectionally associated with eating disorder symptomatology in various quantitative and qualitative studies (43–46).

A less well understood aspect of engagement with the online environment is the one in which the user is subjected to harassment behavior, also known as cyberbullying. Experiencing cyberbullying through social media is considered to have debilitating effects on mental health in general (47), but also significant correlations with body dissatisfaction, negative body perception and lower self-esteem (48,49). The culture of body shaming has sparked a heated debate around whether such an approach can be deemed hazardous for the obesity population as it may well be for the eating disorder populations (50). A quasi-experimental field study showed that fat-shaming events on social media could have putative snowball effects on the anti-fat attitudes in the wider population, the effects increasing with the notoriety of the event (51). Body shaming can masquerade as essential public health messaging or as an essential component in the coach-athlete relationship (52), but essentially may constitute a form of emotional maltreatment (52). While cyberbullying constitutes a form of maltreatment occurring in the online environment, it is possible that its effects may be detrimental for the individual’s global mental health, rather than being ED-specific. However, experiencing body-shaming, regardless of whether the individual is victimized themselves in the event or not, may have specific effects on self-apprehension of weight, shape or appearance, body dissatisfaction or foster unhelpful anti-fat attitudes, which may be strong precursors for the development of EDs. Furthermore, it may promote stigma towards EDs, which can hinder the likelihood of affected individuals accessing timely help, and/or engaging fully with treatment, thereby worsening clinical outcomes (53). For males, it may be even worse, as they need to battle against toxic masculinity and a well-established societal stigma (also present in the medical community) which promotes stereotypes of gender and sexual orientation in relation to those suffering from EDs.

## Trends and developments

**Figure 1:**
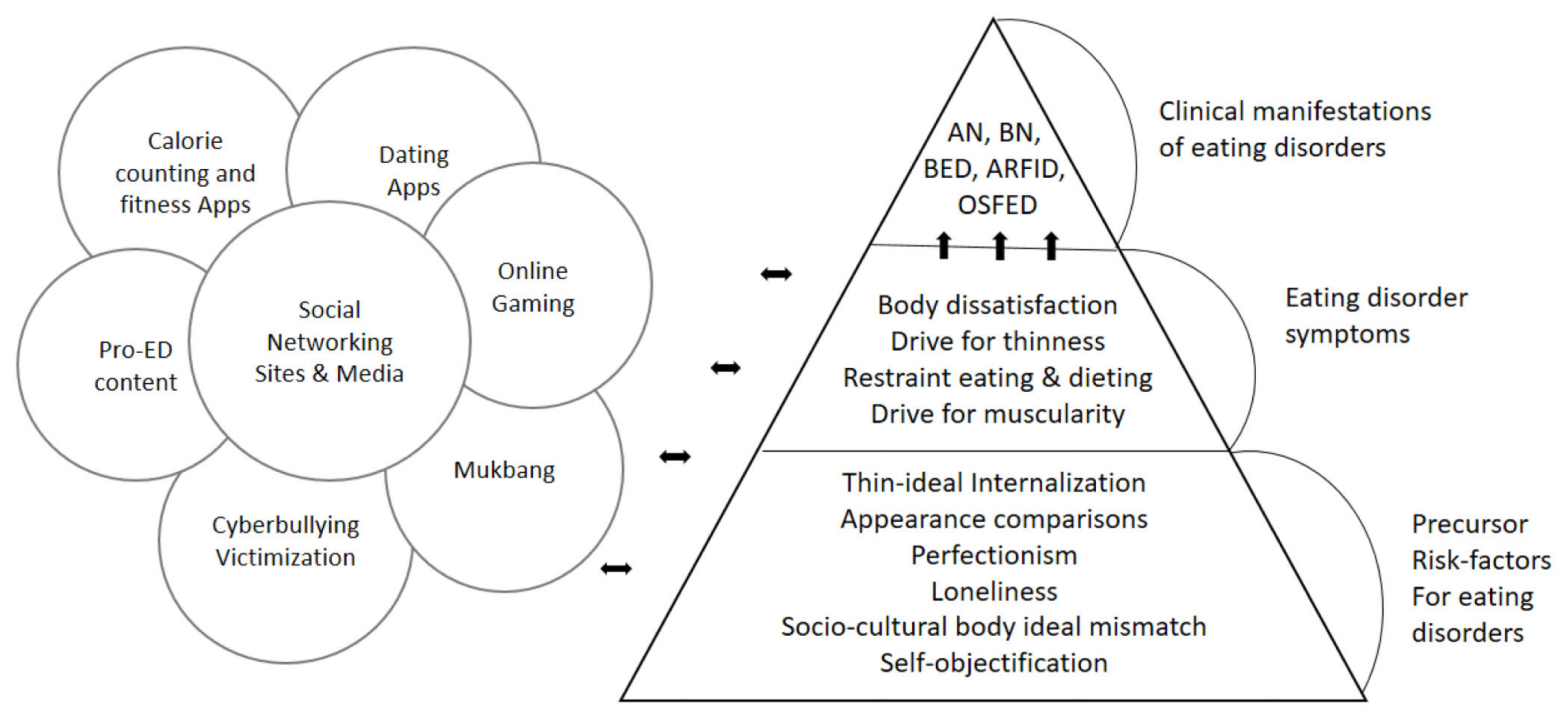
Example illustration of the relationship between multiple facets of internet usage and risk factors, pre-cursors for feeding and eating disorders, as well as eating disorder symptoms.

While a substantial portion of the quantitative literature supports the notion that significant positive correlations exist between various facets of PUI and eating disorder and related psychopathology (19), those observational studies have focused on either precursor risk factors for eating disorders (e.g. the thin-ideal internalization, appearance comparisons, perfectionistic personality traits, loneliness, socio-cultural body ideal mismatch, self-objectification) or eating disorder symptoms which, on their own merits, do not necessarily constitute clinically significant levels of formal mental health disorder (e.g. body dissatisfaction, drive for thinness, dietary restraint, drive for muscularity) (see FIGURE 1).

Equally important is the fact that many of those personality dimensions, risk-factors and symptoms are trans-diagnostic e.g. perfectionism is common in AN but also in obsessive compulsive personality disorder; e.g. body dissatisfaction or drive for muscularity are common in EDs (mainly AN and BN) but also body dysmorphic disorder. Thin-ideal internalization (ITI) is common in AN and BN. The trans-diagnostic nature of those symptoms, together with a paucity of clinical-level ascertainment in the literature does not yet provide the necessary confidence to rigorously confirm causality between hazardous levels of PUI and occurrence of formal EDs. It is furthermore unclear how different facets of ED would be impacted differentially; thus specific PUI behaviors like consumption of ‘thinspiration’ content via SNS might be hazardous towards AN or BN outcomes, while other ED categories e.g. BED or ARFID, where there is less presence of ITI may not be affected. Furthermore, cyberbullying victimization may affect differentially patients at higher BMIs (e.g. BED sufferers) through experiences of body shaming, or those who struggle to comprehend nuances of the online media social context (e.g. those who may have AN and autistic spectrum disorder comorbidity or other deficits in their theory of mind) and might be more vulnerable to cyber-harassment.

Some of the clinical phenotypes of ED are less understood in how the related to PUI. An interesting case series of ARFID and Internet Gaming Disorder (IGD) comorbidity was published (54) suggesting that both ARFID and IGD manifesting as coping strategies (a method of emotional regulation) to those avoidant of emotional distress. The relationship between internet gaming and EDs is less understood, but there are examples in which body shaming and fatness has been used in video games to associate the obese male or female body with evilness, disability, monstrosity and horror (55). Such games are played by hundreds of million users worldwide and have the potential of having a substantial influence in the sociocultural attitudes towards obese body types, potentially adding to weight related stigma (56).

Finally, research has explored how the digital medium can be used to potentially promote a healthier body image; for example, an experimental study showed exposure to parody images of thin-ideal celebrities was associated with higher body satisfaction, when compared to exposure to the actual thin-ideal image (57); self-disclaimers show little effectiveness in altering body image influences (58). Further research is warranted to further understand how technology can be harnessed to promote a healthy relationship with someone’s own body, nutrition and exercise. Identification of vulnerable populations (e.g. via algorithmic/machine learning approaches, with appropriately rigorous statistical methodologies including cross-validation and independent replication) seems to be an important first step, which can then be followed by targeted ED-specific resilience promoting content. Apps, podcasts and health resources already exist aiming to promote ED recovery to those seeking digital solutions to treatment and online delivery of psychotherapy has become the new norm during the COVID-19 pandemic. Most of those processes are currently at a preliminary stage, including how they can translate to real life applications, notwithstanding the major ethical considerations relevant to this process that involve, but not exhaustively, privacy, confidentially and informed consent.

## Insights

While the online environment continues to advance in many profound but also intricate ways, research attention has shifted to other facets of engagement with the online milieu, including, but not exhaustively, the consumption of image-based content through social media and image-based gaming, and even more recently the utilization of fitness-tracking/calorie-tracking applications (Apps), dieting Apps, dating Apps and mukbang streaming consumption (mukbang is further explained below). A recent meta-analysis supported the fact that a variety of PUI facets are important for the understanding of digital hazards for feeding and eating (19). Critically, while SNS have been relatively better studied, the use of activity and fitness tracking Apps, calorie tracking Apps, dating Apps, as well as cyberbullying experiences may be playing a role in the link between PUI and eating disorder and related psychopathology.

## Controversy and debate

Another growing trend has been the investigation towards the understanding of mukbang as an emerging online behavior since 2014. Mukbang (‘muk-bang’ or ‘meokbang’) is Korean – a portmanteau term for ‘eating broadcast’ and usually involves a host preparing and eating typically large amounts of food, while leisurely interacting with an audience in an online streaming platform. By some self-reports, mukbang has been regarded to have both beneficial (e.g. preventing binge eating episodes, reducing loneliness) and negative sequalae (triggering restrained eating or loss of control over eating) on eating disorder symptoms (59•). It is possible that mukbang may be a fun, leisure and intimate experience for a large portion of the population; millions engage with the streaming content daily and professional mukbang streaming is reportedly very profitable for the individual. On the other hand it may create a platform for patients with AN to engage with food without consuming it, thus missing out on essential nutrition leading to self-neglect, or trigger bingeing episodes with untoward consequences (60). It is a well-known fact that ED sufferers, even those at very low BMIs (e.g. AN), are ‘food lovers’ and are generally much more fascinated by food preparation and consumption as compared to the general population. Thus, the fact that mukbang viewing is positively correlated with EDs is not a surprise (61), particularly when the observed behaviors are not ascertained at a maladaptive or clinical level. Anecdotal reports suggest that on occasion mukbang viewing promotes recovery practices (62), therefore it is still debatable whether consuming mukbang is a problematic behavior. Greater research-driven understanding is needed before any causal links are drawn; in approaching the mukbang phenomenon in a confirmatory and atheoretical fashion we risk overpathologizing the behavior, an existing risk in behavioral addictions research in general (63).

## Conclusion

We have discussed here specific facets of engagement with the online environment bear risk for feeding and eating problems viewed largely using dimensional measures in non-clinical settings. Experimental and prospective studies support a potential causal role for the engagement with social media leading to higher levels of body dissatisfaction and internalization of the thin ideal. The role of self-objectification, appearance comparison, impulsivity/compulsivity or the socio-cultural body ideal mismatch is relatively unexplored from a mechanistic perspective, particularly in terms of the interaction of those concepts with a variety of relatively distinct online behaviors (e.g. consumption of pro-ED content, calorie tracking/fitness apps, dating sites, gaming, mukbang streaming or watching, experiencing cyberbullying victimization). More longitudinal and experimental research is required to ascertain specific effects, simulating conditions of the various aspects of potentially hazardous online engagement towards the potential development of eating disorder psychopathology and to further clarify how research findings can best be translated into public health and clinical arenas (e.g. see TABLE 1), in order to optimally protect those who are vulnerable to developing disordered eating symptoms, or experiencing exacerbations thereof.

## Figures and Tables

**Table 1:** Problematic usage of the internet and eating disorders, preliminary recommendations for public health and clinical care

Preliminary recommendations for public health and clinical care	
Public health	Clinical care
Promotion of discussion between stakeholders in regards to whether potentially hazardous online content should be regulated; and if so, how Dissemination of scientific findings into the public domain by appropriate communication, including raising awareness that potentially hazardous content may contribute towards the development of eating and feeding disorders ED-orientated health advice (e.g. ED-helpline, local services access) and supportive messaging to be recommended on image-based online platforms/content to encourage early help seeking for people with EDs Public advice for families caring for sufferers of ED, also experiencing PUI in their home environment Public debate on medical and other considerations relevant for the application of ED-specific resilience building online content (digital medicine / digital public health interventions)	Clinician awareness of how the internet (including PUI) is clinically relevant to understanding and treating EDs,including in eating disorder clinics (e.g. redaction of problematic internet usage may be considered as complementary psychotherapeutic target for those receiving help for their eating disorder together with other established treatment goals) Consideration of screening for eating disorders in newly developing clinics of problematic usage of the internet (e.g. online gaming), particularly if overuse of social media is present Awareness that EDs often coexist with other disorders, including those that can be contributed to by PUI. Exploration of internet related vulnerabilities (e.g. overuse of social media, overconsumption of fitness Apps/ “fitspiration” content) as part of routine ED clinical practice
